# How (some) mammals lost their hair

**DOI:** 10.7554/eLife.84865

**Published:** 2022-12-30

**Authors:** Matthew D Dean

**Affiliations:** 1 Department of Molecular and Computational Biology, University of Southern California Los Angeles United States

**Keywords:** hair, convergent evolution, hairless, regressive evolution, Human, Mouse, Rat, Rhesus macaque, Other

## Abstract

An approach that allows scientists to identify regions of the genome that evolved faster in hairless mammals reveals candidate genetic mechanisms that gave rise to hairlessness.

**Related research article** Kowalczyk A, Chikina M, Clark NL. 2022. Complementary evolution of coding and noncoding sequence underlies mammalian hairlessness. *eLife* **11**:e76911. doi: 10.7554/eLife.76911.

Most mammals have hair which can vary greatly in color, length and texture (Pough et al., 1989). However, there are several so-called ‘hairless’ species – such as whales, walruses, elephants, and humans – that have considerably less hair than other mammals.

Although certain genes connected to the development of hair have been identified, the genetic mechanisms that led to the loss of hair in certain mammals were unknown. One way to uncover candidate genes is to investigate their relative evolutionary rate (RER) in hairless versus haired mammals (Kowalczyk et al., 2019). In theory, the parts of the genome that once contributed to hair growth are free to accumulate more mutations once hairlessness arises. This is because in hairless mammals these regions are no longer used, so mutations in them are not selected against and amass faster.

Now, in eLife, Amanda Kowalczyk, Maria Chikina and Nathan Clark report on the identification of genetic regions that show accelerated evolution in hairless mammals using a computational approach called RERconverge (Kowalczyk et al., 2022). The team (who are based at Carnegie-Mellon University, the University of Pittsburgh and the University of Utah) compared the genomes of 62 mammalian species (including several hairless species) with phylogenetic relationships that indicated hair was lost at least nine times independently. Kowalczyk et al. focused their analyses on around 20,000 protein coding and around 350,000 noncoding regions that were shared among these genomes. Noncoding regions are potentially important players in the regulation of nearby genes.

The analyses of Kowalczyk et al. revealed that 20 protein coding regions and almost 2,000 noncoding regions had evolved significantly faster in hairless versus haired species. Some of the genes identified were known from other research to be involved in hair development, supporting the idea that the RERconverge approach had indeed uncovered regions of the genome relevant to hairlessness. However, most of the genomic sites detected had no known connection to hair growth, adding to the list of genomic regions that may be responsible for hair loss.

By comparing protein coding and noncoding regions, Kowalczyk et al. were able to yield three important insights. First, around ten keratin genes showed accelerated evolution in both coding and noncoding regions, suggesting that both the expression and structure of some proteins have changed in response to hairlessness.

Second, many accelerated noncoding regions did not have rapidly-evolving protein coding sequences nearby. This suggests that the protein coding sequences near these noncoding regions undergo shifts in expression that can affect hair growth, but likely also have roles unrelated to hair growth. This would explain why these protein coding sequences have not undergone accelerated evolution: the proteins they encode need to maintain their structure to perform their other roles, preventing these sequences from rapidly accumulating mutations.

Third, genes with accelerated evolution tended to be expressed in the hair shaft itself, while accelerated noncoding regions were often found near genes expressed in basal cells that give rise to hair (Figure 1). Together, these results show that hairlessness likely evolved through a complex combination of protein coding and noncoding mechanisms.

**Figure 1.**
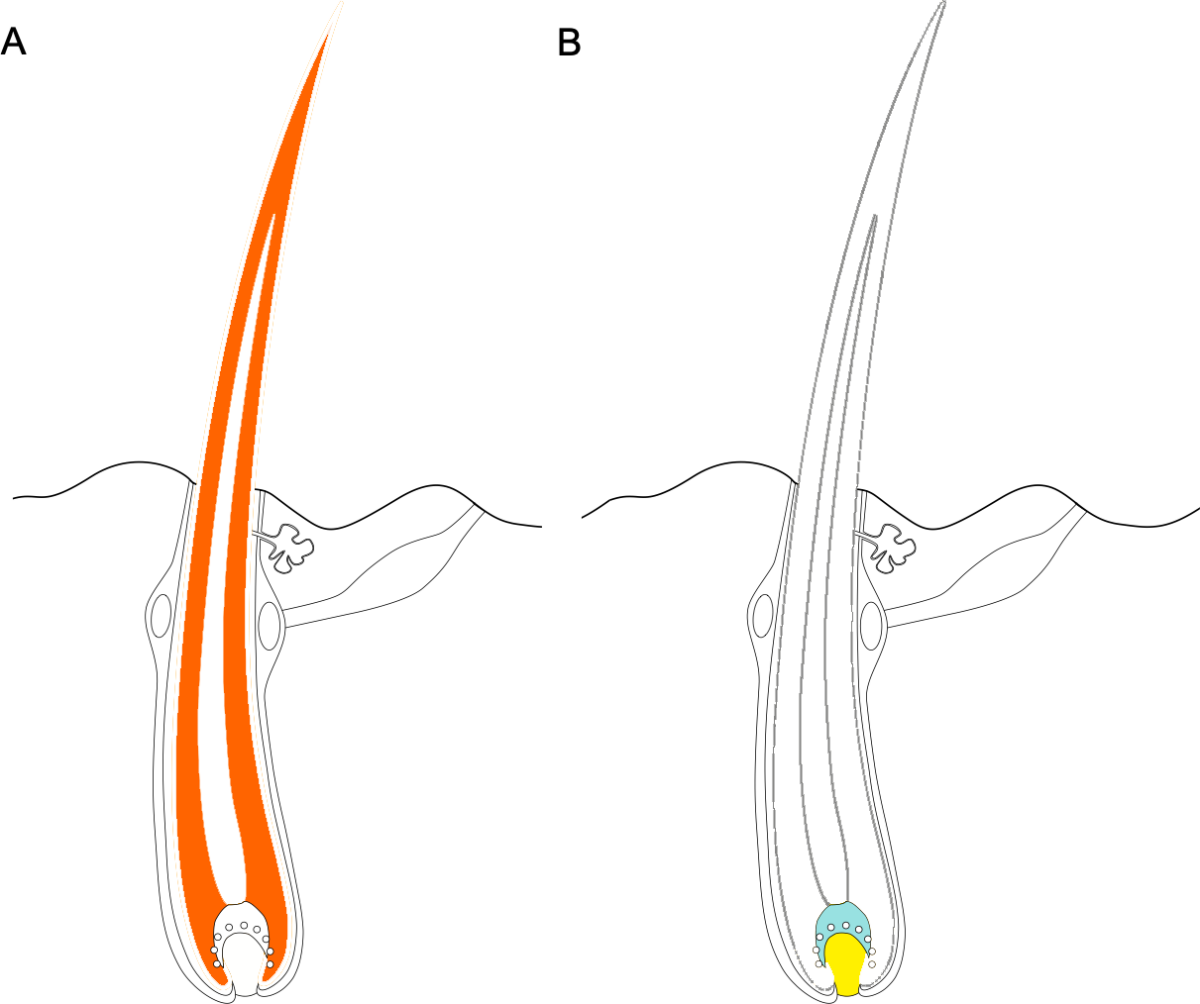
Some regions of the genome - including both coding and noncoding regions - evolve faster in hairless species than in species with hair. (**A**) Protein coding genes that exhibit accelerated evolution in hairless mammals are more highly expressed in the hair shaft, with the cortex of the hair (dark orange area) expressing a more of these genes. (**B**) Genes near accelerated noncoding regions tend to be expressed in the cells at the base of the hair, known as the matrix (shown in blue) and the dermal papilla (shown in yellow). Figure adapted from Kowalczyk et al., 2022.

Evolution has tested countless mutations across individuals and generations, in sample sizes that could never be achieved in laboratory settings. Approaches like RERconverge exploit these evolutionary experiments to identify genetic sequences connected to specific traits by analyzing the speed at which these genomic regions evolve in different species. This is particularly useful to identify regions of the genome involved in the loss a trait (such as the loss of hair), because they will accumulate mutations faster in species that have lost the trait. Indeed, the work by Kowalczyk et al. joins a growing list of studies into the evolutionary genetics of trait loss, which include research into adaptations to aquatic and subterranean lifestyle in mammals (Chikina et al., 2016; Partha et al., 2017). One exciting next step in the study of hair loss will be to genetically modify model organisms like mice to test whether the genomic regions identified by Kowalczyk et al. affect hair growth in the laboratory.
